# Effect of Enhanced Psychological Nursing Combined with Graded Pulmonary Rehabilitation in Elderly Patients with COPD: A Randomized Controlled Trial: Effect of Enhanced Psychological Nursing in Elderly Patients with COPD

**DOI:** 10.31661/gmj.v14i.3874

**Published:** 2025-07-09

**Authors:** Leping Zhang, Jian Cheng, Xiuqin Ma, Qin Lu, Yan Yang

**Affiliations:** ^1^ Department of Respiratory and Critical Care Medicine, Yixing City People’s Hospital, Yixing, Jiangsu Province, China XX

**Keywords:** Chronic Obstructive Pulmonary Disease, Mental Health Intervention, Staged Pulmonary Rehabilitation, Respiratory Function, Life Quality, Patient Compliance

## Abstract

**Background:**

This research aimed to investigate the therapeutic effectiveness of combining structured psychological support with a stepwise pulmonary rehabilitation regimen in older adults diagnosed with stable chronic obstructive pulmonary disease (COPD).

**Materials and Methods:**

In this randomized controlled trial, 120 elderly patients with stable COPD were evenly assigned into two groups (n=60). The control group underwent conventional pharmacologic therapy and standard nursing care. In contrast, the intervention group received additional enhanced psychological interventions along with a progressive pulmonary rehabilitation protocol aligned with the Global Initiative for Chronic Obstructive Lung Disease (GOLD) recommendations. Outcomes assessed before and after the intervention included psychological health indicators, lung function metrics, compliance with treatment, rate of acute exacerbations, six-minute walk distance (6MWD), and quality of life.

**Results:**

While both groups experienced notable improvements in anxiety, depressive symptoms, and psychological resilience, these changes were significantly more favorable in the intervention group (P0.05). Pulmonary function indicators, namely FEV1%, FEV1/FVC, and peak expiratory flow (PEF), showed measurable improvements across both groups, with the intervention group exhibiting more marked progress. Additionally, patients in the intervention group showed higher adherence to treatment, fewer acute exacerbation episodes, and a greater increase in 6MWD over the 12-month follow-up period. Quality of life, assessed via the St. George’s Respiratory Questionnaire, demonstrated more pronounced enhancements in respiratory symptoms, physical activity, and overall disease burden in the intervention group (P0.05 across all domains).

**Conclusion:**

Incorporating targeted psychological support and a graded pulmonary rehabilitation strategy yields substantial benefits in emotional well-being, pulmonary performance, adherence levels, and life quality in elderly COPD patients. This comprehensive care model may serve as an effective approach for sustained disease management in this population.

## Introduction

Chronic obstructive pulmonary disease (COPD) is a long-term pulmonary condition marked by non-reversible airflow obstruction and continuous respiratory symptoms. It commonly leads to substantial limitations in physical function and a decline in life quality, especially in older adults [1]. The natural deterioration of respiratory function with age further complicates disease management, contributing to a higher incidence of acute exacerbations and a less favorable clinical outcome [2].

While pharmacological treatments remain central to COPD management, non-pharmacological interventions, notably pulmonary rehabilitation (PR), have demonstrated substantial benefits [3]. PR is a comprehensive, individualized intervention encompassing exercise training, education, and behavioral modifications aimed at improving physical and psychological conditions [4]. Graded pulmonary rehabilitation (PR), structured based on disease severity levels outlined by the Global Initiative for Chronic Obstructive Lung Disease (GOLD), enables individualized therapeutic strategies aimed at improving physical performance and alleviating dyspnea in geriatric COPD patients [5]. Existing evidence indicates that pulmonary rehabilitation notably enhances functional exercise tolerance—commonly assessed via the six-minute walk test (6MWT)—and contributes to better health-related quality of life in this population [6]. Psychological distress, including anxiety and depression, is prevalent among elderly patients with COPD, adversely affecting treatment adherence and overall outcomes [7]. Enhanced psychological nursing interventions, focusing on individualized psychological support, have been shown to alleviate emotional disturbances, thereby improving engagement in rehabilitation programs [8]. Integrating psychological care with graded pulmonary rehabilitation may offer synergistic benefits, addressing both physical and emotional aspects of COPD [9].

Although the advantages of both graded pulmonary rehabilitation and psychological therapies are well established individually, few studies have investigated the synergistic impact of combining these approaches in older adults with COPD. This study aims to evaluate the clinical effectiveness of an integrated approach—enhanced psychological nursing combined with GOLD-guided graded pulmonary rehabilitation—in improving psychological and physical outcomes in elderly patients with COPD, thereby providing evidence for more holistic and personalized clinical interventions in this vulnerable population.

## Materials and Methods

**Figure-1:**
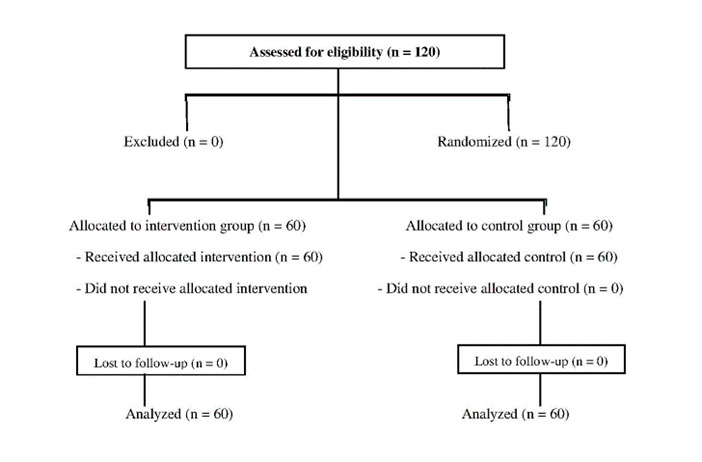
Figure 1. CONSORT Flow Diagram.

### Study Design and Study Settings

This randomized controlled trial was conducted at Yixing City People’s Hospital between March 2023 and March 2024. A total of 120 elderly patients diagnosed with stable chronic obstructive pulmonary disease (COPD) were recruited for the study. Participants were randomly divided into two groups—control and intervention—each consisting of 60 patients, using a random number table for allocation. The study protocol received approval from the Ethics Committee of Yixing City People’s Hospital (Approval No. BER-YXPH-2024050), and all subjects signed informed consent forms prior to participation. This trial was prospectively registered in the Chinese Clinical Trial Registry (ChiCTR) under the registration number ChiCTR2200067819, Figure-1 shows the CONSORT flow diagram of participant enrollment, allocation, follow-up, and analysis

### Sample Size Calculation

The sample size was determined based on preliminary findings, anticipating a minimum 15% difference in FEV1% improvement between groups. Setting the significance threshold (α) at 0.05 and statistical power (1-β) at 80%, the estimated sample size was 52 participants per group. To account for an estimated 15% attrition rate, the enrollment target was increased to 60 subjects per group to maintain adequate study power.

### Participants

Inclusion criteria:

• Diagnosed with COPD according to the Diagnosis and Treatment Guidelines for Chronic Obstructive Pulmonary Disease (Revised 2021 Edition) [10].

• Age ≥ 60 years

• Stable phase of COPD confirmed by examination

• Conscious with no cognitive or communication impairment

• Signed informed consent after understanding the study objectives and methods

Exclusion criteria:

• Severe heart, liver, kidney, or motor system diseases

• Respiratory diseases other than COPD such as bronchial asthma or bronchiectasis

• Cognitive, communication, or eating disorders

• Mental disorders, senile dementia, or malignant tumors

Baseline demographic and clinical characteristics such as gender, age, disease duration, smoking history, dyspnea levels, and education were comparable between groups (P>0.05).

### Interventions

Control group: Received routine treatment including pharmacological therapy (tiotropium bromide, formoterol, inhaled corticosteroids for moderate to severe cases, ambroxol, N-acetylcysteine), non-pharmacological interventions (smoking cessation advice, environmental control), and routine care (health education, dietary guidance, respiratory function training, and low-intensity aerobic exercise).

Intervention group: Received all the control group interventions plus enhanced psychological nursing and graded pulmonary rehabilitation based on GOLD criteria. Psychological nursing included a structured program based on SCL-90 scoring, individualized psychological support, cognitive-behavioral therapy, family involvement, multimedia education, and positive psychotherapy. Pulmonary rehabilitation intensity was tailored according to COPD severity (GOLD levels 1 to 3).

Both interventions lasted for 12 weeks.

At the intervention stage, both groups received standard COPD pharmacologic and non-pharmacologic care. However, the intervention group received additional enhanced psychological nursing care. A detailed comparison of interventions between the two groups is provided in Table-1.

### Blinding

Due to the characteristics of the psychological and rehabilitation interventions, blinding of participants and care providers was not feasible. However, to reduce potential bias, the individuals responsible for outcome assessment and data analysis remained blinded to group allocation throughout the trial.

### Outcome Measures

1. Psychological status: Evaluated using the Hamilton Anxiety Scale (HAMA), Hamilton Depression Scale (HAMD), and Connor-Davidson Resilience Scale (CD-RISC) at baseline and 12 weeks after intervention.

2. Pulmonary function: Forced expiratory volume in one second (FEV1%), FEV1/forced vital capacity (FVC) ratio, and peak expiratory flow (PEF) were measured three times using a portable spirometer before and after the intervention.

3. Treatment adherence: Classified as full compliance, partial compliance, or non-compliance; overall compliance rate was calculated post-intervention.

4. Frequency of acute exacerbations: Recorded over 12 months before and after the intervention.

5. Six-minute walk distance (6MWD): Conducted by a trained therapist in a standardized corridor environment.

6. Quality of life: Assessed using the St. George’s Respiratory Questionnaire (SGRQ) before and 12 weeks after the intervention.

### Statistical Analysis

Data were analyzed with IBM SPSS Statistics for Windows, version 26 (IBM Corp., Armonk, N.Y., USA) Continuous variables are expressed as mean ± standard deviation (±SD). Between-group comparisons used independent sample t-tests, while paired t-tests assessed within-group changes pre- and post-intervention. Categorical variables were analyzed via chi-square (χ²) tests, and ordinal data were evaluated using rank-sum tests. A P-value less than 0.05 was considered statistically significant.

## Results

**Table:** Table1. Comparison of interventions between the control and intervention groups.

**Intervention Type **	**Control Group **	**Intervention Group **
**Pharmacological Treatment **	Tiotropium bromide, formoterol, ICS for moderate to severe cases, ambroxol, N-acetylcysteine	Same as control group
**Non-pharmacological Management **	Smoking cessation advice, environmental factor control	Same as control group
**Routine Nursing Care **	Basic health education, medication adherence guidance, follow-up of symptoms	Same as control group
**Enhanced Psychological Nursing **	Not provided	Comprehensive psychological support including motivational interviewing, pulmonary rehabilitation, and regular cognitive-behavioral assessment

**Table:** Table2. Comparison of scores related to psychological states between two groups(x¯- ±s,score)

**groups**	**HAMA**		**HAMD**		**CD-RISC**	
	**pre-intervention**	**post-intervention**	**pre-intervention**	**post-intervention**	**pre-intervention**	**post-intervention**
**Observation Group** **（** **n=60** **）**	21.86±1.30	15.89±1.07 ^*^	32.88±2.17	21.58±2.10 ^*^	49.86±11.25	71.38±11.92 ^*^
**Control group** **（** **n=60** **）**	22.15±1.35	17.97±1.10 ^#^	33.09±2.12	25.71±2.23 ^#^	50.14±12.07	57.82±12.30 ^#^
***t***	1.199	10.499	0.536	10.444	0.131	6.132
***P***	0.233	＜0.001	0.593	＜0.001	0.896	＜0.001

Note: Comparison with pre-intervention, ^*^P<0.05, #P< 0.05; **HAMA:** Hamilton Anxiety Scale; **HAMD:** Hamilton Depression Scale; **CD-RISC:** Psychological Resilience Scale

### Psychological Status Comparison

No significant differences were found between groups in baseline HAMA, HAMD, and CD-RISC scores (P > 0.05). Both groups demonstrated significant improvements after intervention, with reductions in HAMA and HAMD scores and increases in CD-RISC scores compared to baseline (P < 0.05). Notably, the intervention group showed significantly better psychological outcomes than the control group at follow-up (P < 0.05, Table-1). These results are summarized in Table-2.

### Pulmonary Function Comparison

At baseline, pulmonary function parameters (FEV1%, FEV1/FVC, PEF) were similar across groups (P > 0.05). Following 12 weeks of treatment, significant improvements were observed in both groups (P < 0.05), with the intervention group exhibiting more pronounced enhancement in lung function metrics compared to controls (P < 0.05, Table-3).

### Treatment Compliance Comparison

The treatment compliance rate in the intervention group reached 96.67%, significantly exceeding the 85.00% compliance observed in the control group (χ² = 4.904, P = 0.027), suggesting superior adherence among those receiving the combined intervention (Table-4).

### Acute Exacerbations and 6MWD

Prior to treatment, no significant differences existed between groups regarding acute exacerbation counts or 6MWD (P > 0.05). Both groups improved significantly at 12-month follow-up; however, the intervention group experienced fewer acute exacerbations and greater gains in 6MWD compared to controls (P < 0.001), underscoring the intervention’s effectiveness (Table-5).

### Quality of Life (SGRQ Scores)

Baseline SGRQ scores—covering respiratory symptoms, activity limitation, disease impact, and total score—were comparable between groups (P > 0.05). Post-intervention, all domains improved significantly in both groups (P < 0.05), with the intervention group achieving superior outcomes across all aspects (P < 0.05, Table-6).

## Discussion

**Table:** Table3. Comparison of two groups of lung function indicators(x¯ ±s)

**groups**	**FEV1%**		**FEV1/FVC** **（** **%** **）**		**PEF** **（** **L/s** **）**
	**pre-intervention**	**post-intervention**	**pre-intervention**	**post-intervention**	**pre-intervention**	**post-intervention**
**Observation Group** **（** **n=60** **）**	45.74±4.59	57.94±5.87 ^*^	54.83±8.08	70.92±7.99 ^*^	0.34±0.08	0.54±0.12 ^*^
**Control group** **（** **n=60** **）**	46.08±5.11	53.29±4.12 ^#^	55.20±7.85	66.42±9.11 ^#^	0.33±0.07	0.44±0.10 ^#^
***t***	0.383	5.022	0.254	2.877	0.729	4.959
***P***	0.702	＜0.001	0.800	0.005	0.468	＜0.001

Note: Comparison with pre-intervention,^*^P<0.05, #P< 0.05; **FEV1%:** The percentage of forced expiratory volume in 1 second to the expected value; **FEV1/FVC:** forced expiratory volume in 1 second/forced vital capacity; **PEF:** peak expiratory flow

**Table:** Table4. Comparison of treatment compliance between two groups n(%).

**groups**	**Fully comply**	**Partial compliance **	**non-compliance**	**Overall compliance rate **
**Observation Group ** **（** **n=60** **）**	40（66.67）	18（30.00）	2（3.33）	58（96.67）
**Control group** **（** **n=60** **）**	35（58.33）	16（26.67）	9（15.00）	51（85.00）
***χ ^2^***				4.904
***P***				0.027

**Table:** Table5. Comparison of acute exacerbation frequency between two groups and 6MWD(x¯ ±s)

**groups**	**Number of acute exacerbations (times)**		**6MWD** **（** **m** **）**	
	**pre-intervention**	**post-intervention**	**pre-intervention**	**post-intervention**
**Observation Group** **（** **n=60** **）**	2.08±0.92	0.48±0.22 ^*^	385.72±78.93	451.26±66.20 ^*^
**Control group** **（** **n=60** **）**	2.12±1.04	1.35±0.52 ^#^	371.28±75.88	395.20±63.34 ^#^
***t***	0.223	11.935	1.022	4.740
***P***	0.824	＜0.001	0.309	＜0.001

Note: Comparison with pre-intervention,^*^P<0.05, #P< 0.05; **6MWD:** 6-minute walking test distance

**Table:** Table6. Comparison of SGRQ scores between two groups(x¯ ±s,score)

**groups**	**Respiratory symptoms **		**Disease impact **		**Activity ability **		**Total score**	
	pre-intervention	post-intervention	pre-intervention	post-intervention	pre-intervention	post-intervention	pre-intervention	post-intervention
**Observation Group ** **（** **n=60** **）**	59.76±8.22	38.14±9.30 ^*^	36.24±3.71	22.57±3.68 ^*^	46.93±7.11	62.83±8.24 ^*^	48.12±3.82	39.75±4.98 ^*^
**Control group** **（** **n=60** **）**	60.15±6.81	46.86±7.18 ^#^	36.77±4.78	26.53±3.56 ^#^	47.09±7.60	57.32±7.66 ^#^	48.20±3.93	43.69±3.65 ^#^
***t***	0.283	5.749	0.679	5.991	0.119	3.794	0.113	4.943
***P***	0.778	＜0.001	0.499	＜0.001	0.905	＜0.001	0.910	＜0.001

Note: Comparison with pre-intervention,^*^P<0.05, #P< 0.05

This study evaluated the combined effect of enhanced psychological nursing and graded pulmonary rehabilitation on mental health, lung function, treatment adherence, and quality of life in elderly patients with chronic obstructive pulmonary disease (COPD).

Our findings indicate that the intervention group showed significant improvements in all assessed outcomes compared to the control group, supporting our hypothesis that a comprehensive, individualized intervention provides superior benefits for elderly COPD patients [10].

These findings are consistent with earlier studies that have highlighted the benefits of pulmonary rehabilitation in enhancing exercise tolerance and health-related quality of life for individuals with COPD. For instance, Katsura et al. demonstrated that a 6- to 12-week outpatient pulmonary rehabilitation regimen significantly increased gait speed and decreased frailty among patients with chronic respiratory diseases, including COPD [11]. Likewise, Stoffels et al. observed significant improvements in physical performance metrics following both inpatient and outpatient pulmonary rehabilitation interventions in COPD populations [12]. Additionally, multiple studies have substantiated that structured exercise programs combined with respiratory muscle training improve functional capacity and contribute to lower rates of rehospitalization in patients with moderate to severe COPD [13][14].

Furthermore, the integration of psychological interventions has been shown to enhance pulmonary rehabilitation outcomes. Bove et al. demonstrated that psychoeducational interventions based on cognitive-behavioral therapy (CBT) effectively reduced anxiety and increased patients’ sense of mastery in severe COPD cases [15]. Jordan et al. also highlighted that structured exercise incorporated into multicomponent interventions improves health-related quality of life and decreases dyspnea severity [16]. These findings are echoed in a study by Yohannes et al., who found that CBT and tailored counseling reduced depressive symptoms and improved emotional well-being in older adults with COPD [17].

Contrastingly, some studies report limited benefits when psychological interventions are applied alone. Lee et al. found no significant differences in coping, self-efficacy, or depressive symptoms between COPD patients receiving nurse-led problem-solving therapy and those receiving usual care [18]. However, subgroup analyses indicated that patients with clinical depression experienced improvements, suggesting psychological interventions may be most effective when tailored to patient-specific conditions. This is supported by a randomized trial by Kunik et al., in which patients with baseline anxiety or depression benefited more from integrated behavioral care [19].

Moreover, recent meta-analyses emphasize that combining psychological support with pulmonary rehabilitation not only improves mental health outcomes but also enhances physical endurance and reduces healthcare utilization [20][21].

In summary, our results underscore the value of a comprehensive and personalized treatment strategy that integrates both physical rehabilitation and psychological support, consistent with the recommendations of the Global Initiative for Chronic Obstructive Lung Disease (GOLD) guidelines [22]. By simultaneously targeting the physiological and psychological aspects of COPD, this approach can lead to marked improvements in patient outcomes and overall quality of life.

## Conclusion

This study demonstrates that enhanced psychological nursing combined with graded pulmonary rehabilitation yields significant improvements in psychological well-being, pulmonary function, treatment adherence, and health-related quality of life in elderly patients with COPD. Compared to routine care, this individualized and comprehensive intervention led to superior outcomes across all measured parameters, including reduced anxiety and depression levels, improved resilience, greater pulmonary function indices, higher treatment compliance, reduced frequency of acute exacerbations, increased physical endurance (6MWD), and better SGRQ scores. These findings underscore the critical value of integrating psychological support into physical rehabilitation programs for chronic respiratory diseases. Given the complex and multidimensional challenges faced by elderly COPD patients, a holistic treatment strategy that addresses both physiological and psychological aspects is not only justified but necessary. This approach aligns with current international guidelines, such as the GOLD recommendations, and should be considered for broader implementation in routine clinical practice to enhance overall disease management and patient-centered outcomes. Future research with larger sample sizes and multi-center designs is recommended to validate and generalize these findings.

## Conflict of Interest

The authors declare no conflict of interest.
